# Monitoring of bacterial pathogens at workplaces in power plant using biochemical and molecular methods

**DOI:** 10.1007/s00420-017-1197-z

**Published:** 2017-01-25

**Authors:** Anna Ławniczek-Wałczyk, Małgorzata Gołofit-Szymczak, Marcin Cyprowski, Agata Stobnicka, Rafał L. Górny

**Affiliations:** Biohazard Laboratory, Department of Chemical, Aerosol and Biological Hazards, Central Institute for Labour Protection–National Research Institute, Czerniakowska 16 Street, 00-701 Warsaw, Poland

**Keywords:** Molecular typing, RAPD-PCR, Occupational exposure, Bacteria, Source tracking

## Abstract

**Purpose:**

The aim of this study was to characterize the ways of spreading of the most common bacterial species isolated from workers as well as from the air and raw materials at the workplaces in power plant utilizing biomass sources. To monitor microbial transmission and identify the source of contamination in the working environment, a combination of molecular and biochemical methods was applied.

**Methods:**

The study was carried out at workplaces in power plant utilizes biomass as a main fuel source. At each of the studied workplaces, bioaerosol particles were collected on sterile Teflon filters using personal conical inhalable samplers (CIS), and biomass samples (straw pellets and briquettes, corn briquettes, sunflower pellets and wood chips) were directly taken from their storage places. Simultaneously with that, the swab samples from the hands of ten workers and their used respiratory masks (of FFP2 class) were also collected after the work shift to evaluate individual workers’ microbial contamination. In all collected samples, total bacterial concentrations were assessed and the most common microbial isolates were identified to the species level using both biochemical (API tests) and molecular polymerase chain reaction (PCR), followed by random amplification of polymorphic DNA (RAPD) typing methods.

**Results:**

The mean concentrations of culturable bacteria in the air and in biomass samples at the studied workplaces were high, i.e. 1.2 × 10^6^cfu/m^3^ and 3.8 × 10^4^cfu/g, respectively. The number of bacteria in the swab and mask samples also reached a high level of 1.4 × 10^4^ cfu/ml and 1.9 × 10^3^ cfu/cm^2^, respectively. Among the most frequently isolated microorganisms from all types of samples were Gram-positive bacteria of the genus *Bacillus* and *Staphylococcus xylosus*. 37 bacterial strains belonging to the genus *Bacillus* (*B. licheniformis* 8, *B. pumilus* 15 and *B. subtilis* 4) and *Staphylococcus* (10) were genotyped by the RAPD-PCR method. Based on RAPD-PCR analyses, the genomic similarity among 19 *Bacillus* strains isolated from biomass, air, protective mask and hand samples as well as 6 *S. xylosus* strains isolated from air, mask and hand samples exceeded 80%.

**Conclusion:**

This study demonstrated that biomass is the primary source of bacteria at power plant workplaces. These results also revealed that biomass-associated bacteria can be easily transferred to workers’ hands and mask during their routine activities. To improve health protection at the workplaces, adequate training courses on hand hygiene and how to use and remove respiratory masks correctly for workers should be introduced as a key element of the prevention strategy. From the occupational point of view, the PCR-based methods seem to be an efficient tool for a fast and precise typing of bacterial strains isolated from different sources in the occupational environment. Such methods may help to implement appropriate prophylactic procedures and minimize transmission of infectious agents at workplaces.

## Introduction

It is well known that poor hygiene creates favorable conditions for pathogen transfer in occupational settings. Bacterial pathogens are usually transmitted at the workplaces via workers’ hands, clothes as well as contaminated equipment. Previous investigations showed that numerous harmful bacteria can persist on dry surfaces for months (Kramer et al. 2006; Stepanović et al. 2008). Hence, a single hand contact with contaminated surfaces like work tables, doors or other equipment that have been in contact with an infected person may result in pathogen transfer (e.g. to a secondary surface and/or to other uninfected individuals). Microbial pathogens as well as their cell fragments and/or toxins can be also released during normal human activity from contaminated surfaces to the air and may pose a hazard to exposed individuals (Kramer et al. 2006; Otter and French 2009; Qian et al. 2014; Scott 2013).

Proper environmental control of microbial contamination in the working environment should be based not only on the identification of high-risk pathogens, but also focus on the qualitative characterization of the prevailing microbial communities. Such monitoring should allow the detection of infection sources, microbial pathways and control of diversity of individual microbial strains both in the workplace as well as in individual workers. Modern biochemical tests consist of miniaturized strips with microtubes containing dehydrated substrates and enabling comprehensive identification using online databases. These tests allow obtaining the biochemical profiles of specific microorganisms within 6–24 h after inoculation. Nowadays, such biochemical methods make identification simple and ensure the repeatability of the obtained results. It should also be pointed out that calculation of bacterial colonies growing on agar plates (in colony-forming units, cfu) does not require great skill, but analyzing the colony morphology on different media (size, shape, color, texture, elevation, margin, etc.), as well as microscopic characterization of the microbial cells and components, requires deeper knowledge and years of laboratory experience (Jensen and Schafer 1998). In turn, the molecular methods give significant advantages over biochemical methods because they are not dependent on bacterial culturability. For the last 35 years, PCR-based methods have completely revolutionized the area of microbiology, especially clinical bacteriology. For example, rapid detection and differentiation of harmful bacterial isolates during outbreaks can not only minimize transmission, but also adjust control practices and enhance therapeutic treatment (Al-Mohri et al. 2008; González-Rey et al. 2003; Su et al. 2012; van Hal et al. 2007). However, there is no one universal method allowing identification and differentiation of microbial communities in clinical and environmental samples. A vast majority of the hitherto published reports regarding culture-based or molecular detection of microbial species in the occupational environments has been focused on the high-risk pathogens in health-care settings (Klingenberg et al. 2001; Ranjbar et al. 2014; Weber et al. 2013). A little attention has been so far given to microbial source tracking at workplaces in other occupational environments such as, e.g. the energy industry. During the last 15 years in Europe, the use of biomass (the biodegradable fraction of products/waste from agriculture, forestry and related industries) as main fuel to generate heat and electricity has been significantly increased (Directive 2009/28/EC). The consequence of this situation was the creation of many new workplaces at which the exposure to harmful biological agents has been of a great importance. Previous studies have shown that handling and processing of biomass create a risk for workers due to their exposure to harmful bacterial and fungal agents released from this organic source (Ławniczek-Wałczyk et al. 2012; Madsen 2006, 2009). This problem still seems to be important and requires broadening of our knowledge regarding the identification of harmful biological agents, their routes of transmission at workplaces as well as the ways to reduce such exposure. Despite the fact that in the scientific literature many studies about detection of various groups of pathogens are available, there is a visible lack of a comprehensive approach to qualitative and quantitative evaluation of microbiological contamination in the work environment based on advanced analytical techniques. There are also very few studies concerning environmental transmission of pathogens as well as comparing the relationship between microorganisms isolated from contaminated materials presented in the workplace (e.g. waste, biomass, litter, product) and isolates from the breathing zone of the employee (bioaerosol samples) or from hands of workers (Orsini et al. 2002; Rastogi and Sani 2011; Scott 2013; Vela et al. 2012; Zeinali et al. 2015).

In this study, the ways in which the most common bacterial species isolated from workers as well as from the air and raw materials at the workplaces in power plant utilizing biomass sources were characterized. To monitor microbial transmission and identify the source of contamination in the working environment, a combination of molecular and biochemical methods was applied.

## Materials and methods

The study was carried out at workplaces in the power plant utilizes biomass as a main fuel source, in summer 2014. The tested power plant is one of the largest in Poland with a generation capacity of 205 MW. It is equipped with new circulating fluidized bed (CFB) boiler which allows to produce 100% energy from biomass (80% from wood chips and 20% from agricultural waste, such as straw pellets and briquettes, corn briquettes, sunflower pellets). The sampling points were located at workplaces associated with reloading, separation, sizing, storage and conveyor’s transport of biomass (two sampling points). At each of the workplaces, bioaerosol and biomass samples, used respiratory mask and swab samples from hands of workers were collected. The bioaerosol samples were collected during work shift on sterile Teflon filters using personal conical inhalable samplers (CIS) with pumps (Casella CEL, Bedford, Great Britain) at a flow rate of 4l/min for 3 h. After sampling, the filters were placed in sterile test tubes and transported to the laboratory. After filter extraction in 10 ml of saline containing 0.05% of Tween 80 (Sigma-Aldrich Chemie GmbH, Steinheim, Germany), the supernatant was spread in duplicate on Petri plates with appropriate microbiological media: for mesophilic bacteria, TSA agar with 5% sheep blood (Tryptone Soya Agar, BTL, Łódź, Poland); and for Gram-negative bacteria, EMB agar (eosin–methylene blue agar, BTL). The microbiological media contained cycloheximide (50 mg/l, BTL) for inhibiting the growth of saprophytic fungal microorganisms. The plates were then incubated for 1 day at 37 °C + 3 days at 22 °C. After incubation, the bioaerosol concentration was calculated as colony-forming units per 1 m^3^ (cfu/m^3^). The bioaerosol samples were taken twice at each of the sampling location and an additional two samples were collected 80 m from the power plant as outdoor background. The five types of biomass were taken from their storage places: straw pellets and briquettes, corn briquettes, sunflower pellets and wood chips. In this study, three 1 kg samples of each biomass type were collected. Each time, 10 g of collected raw material was transferred to flasks containing 90 ml of saline with Tween 80 and shaken for 1 h. After sedimentation of solid biomass particles, serial dilutions were prepared from the supernatant and spread in duplicate on blood TSA and EMB agar plates. The concentration of bacteria in biomass was presented as cfu/g. Simultaneously with the above-described measurements, two swab samples from the hands of ten workers and from the two used respiratory masks (of FFP2 class) were also collected after the work shift to evaluate microbial contamination. Sterile nylon swabs (Quantiswab, COPAN, Brescia, Italy) were used to take samples from the palm, fingers and between finger surfaces from each worker’s hand. The swabs were shaken for 15 min in 2 ml sterile saline buffer (COPAN) and the resulting suspensions were spread in duplicate on agar plates. The types of agar and incubation conditions were the same as for bioaerosol samples. Bacterial counts were expressed as cfu per 1 ml of extraction buffer. The microbial contamination level of workers’ mask was assessed by the modified Brosseau et al.’s method (1997). From each collected mask, a piece of 10 cm^2^ was cut and extracted with 30 ml of sterile saline containing 0.05% of Tween 80 (Sigma-Aldrich Chemie GmbH). The resulting samples were then shaken for 1 h. After that, serial dilutions were prepared from the supernatant and spread in duplicate on the TSA and EMB agars. The agar plates were incubated at the same condition as bioaerosol samples. The concentration of bacteria in mask samples was presented as cfu per 1 cm^2^. From all samples, the isolated bacterial colonies were identified to the genus and/or species level based on their morphology, microscopic structure and biochemical reactivity using API tests (bioMérieux, Marcy-I’Etoile, France). The collected data of culture-based analysis were statistically processed using the analysis of variance (ANOVA) followed by Scheffe’s test. A 0.05 level of significance was used for all analyses. All data were analyzed using Statistica (data analysis software system) version 10 (StatSoft, Inc., Tulsa, OK, USA).

The most prevalent microbial isolates were additionally analyzed by molecular methods (polymerase chain reaction (PCR), followed by random amplification of polymorphic DNA (RAPD) typing. The selected strains isolated from various samples and analyzed by RAPD-PCR are shown in Table 1. Genomic DNA from each isolate was extracted using EXTRACTME BACTERIA kit (BLIRT SA, Gdańsk, Poland) according to the protocol recommended by the manufacturer. The concentration and purity of DNA were spectrophotometrically assessed and then stored at −20 °C for further analyses.

**Table 1:** The bacterial strains selected for RAPD-PCR analysis

Sources of the sample	Microorganisms			
	*Staphylococcus xylosus*	*Bacillus licheniformis*	*Bacillus pumilus*	*Bacillus subtilis*
Bioaerosol	F4, F15, F19	–	F5, F12, F20, F21	–
Biomass	B2	B8, B31, B35, B39	B13, B17, B37, B40	B4.1, B45
Respiratory mask	M5, M13, M14	M7, M22	M8.1, M19, M24, M21	–
Swab samples from hands	W1, W2, W6	W4, W19	W13, W18, W22	W16, W24

The tested strains belonged to *Bacillus* (27) and *Staphylococcus* (10) genera. RAPD-PCR analysis was performed in a thermocycler (Applied Biosystems, Foster City, USA). All *Bacillus* species were analyzed using a single primer of arbitrary nucleotide sequence Bac1—5′ AGCAGCGTGG 3′ (Cocconcelli et al. 1997). For *Staphylococcus xylosus* analysis, Xyl2—5′ GAGCGGCCAAAGGGAGCAGAC 3′ primer was used (Vela et al. 2012). Primers had a G + C content ranging from 66 to 70%. All reaction mixtures were carried out in a final volume of 25 µl and contained: 1 µl of genomic DNA, 0.5 µl of polymerase TaqNova 2U (BLIRT S.A.), 1 µm of a primer (10 µM), 2.5 µm of each dNTPs (8 mM), 50 mM MgCl_2_, 2.5 µm of 10× TaqNova buffer (BLIRT S.A) and 16 µm of water. The temperature profile of the reaction with the Bac1 primer was as follows: (a) initial denaturation at 95 °C for 3 min, (b) denaturation at 94 °C for 30 s, (c) primer annealing at 45 °C for 1 min, (d) primer extension at 72 °C for 2 min, (e) final extension at 72 °C for 5 min and 4 °C even after the reaction. Points b–d were repeated 35 times. For the Xyl2 primer, the reaction conditions were as following: (a) initial denaturation at 95 °C for 3 min, (b) denaturation at 94 °C for 30 s, (c) primer annealing at 50 °C for 1 min, (d) primer extension at 72 °C for 2 min, (e) final extension at 72 °C for 5 min, 4 °C for ever after the reaction. Points b–d were repeated 35 times. The PCR products were electrophoretically separated on 6% polyacrylamide gel (SE660, Hoefer Inc., Holliston, MA, USA). Separation was achieved at a voltage of 8 V/cm of the gel in 1× TBE buffer for 3 h and 40 min. After completion of the separation, the gel was stained in a bath in an ethidium bromide solution (0.5 mg/l of water). The PCR products were visualized under UV light using a transilluminator at a wavelength of 302 nm. The RAPD patterns were scored on the basis of the presence or absence of the band in the fingerprints. To establish the relationships among isolates, the RAPD-PCR profile grouping was performed with the FPQuest (BIO-RAD, Hercules, CA, USA) software package. The similarities between the fingerprints were calculated using the Pearson product–moment correlation coefficient, and the fingerprints were grouped according to their similarities by use of the UPGMA (unweighted pair group method using arithmetic averages) algorithm. In this study, all types of samples were taken and analyzed in duplicate (bioaerosol, swabs and respiratory masks) or triplicate (biomass). The number of collected samples are presented in Table 2.

**Table 2:** The concentration of bacteria in different types of samples collected at the power plant

Types of samples (number of collected samples)	Bacterial concentrations		
	Arithmetic mean	Standard deviation	Range
Bioaerosol [cfu/m^3^]			
Reloading (2)	427143	23234	410714–443571
Separation and sizing (4)	526683	252820	237500–705882
Conveyor’s transport (4)	2481192	1927193	56250–4119601
Storage (2)	256410	184936	125641–387179
Background (2)	536	84	476–595
Biomass [cfu/g]			
Wood chips (3)	105000	18457	90000–129000
Straw pellets (3)	4350	212	4200–4500
Straw briquettes (3)	7500	424	7200–7800
Corn briquettes (3)	5450	495	5100–5800
Sunflower pellets (3)	305	35	280–330
Respiratory masks [cfu/cm^2^] (20)	1888	577	1050–2950
Swabs from hands [cfu/ml] (20)	14337	13991	312–35600

## Results

### Culture-based analysis

The concentrations of bacteria in different types of samples collected at the studied power plant are presented in Table 2. The bacterial aerosol concentrations ranged from 5.6 × 10^4^ to 4.1 × 10^6^cfu/m^3^. The highest concentrations were recorded at workplaces involved in the conveyor’s transport of biomass, while the lowest were noted at background sampling points (Scheffe test: *p* < 0.0001–*p* < 0.01). The concentrations of bacteria in biomass samples ranged from 2.8 × 10^2^ to 1.3 × 10^5^cfu/g. The highest bacterial content was noted in wood chips; the lowest contamination was observed in the sunflower pellets (Scheffe test: *p* < 0.0001). The number of bacteria in the swab and respiratory mask samples of power plant workers also reached a high level of 1.5 × 10^4^cfu/ml and 1.9 × 10^3^cfu/cm^2^, respectively.

The detailed characteristics of all identified bacteria ar given in Table 3. A total of 24, 26, 18 and 22 bacterial species were identified in bioaerosol, biomass, mask and swab samples, respectively. The most numerous bacterial group in bioaerosol and biomass samples was Gram-positive bacilli constituting 39.8–43.9% of all isolated strains, followed by Gram-positive cocci (*Staphylococcus, Micrococcus* and *Kocuria*) and Gram-positive rods constituting 5–26 and 12–25.7% of isolated microbiota, respectively. Gram-positive cocci predominated in masks and swab samples and accounted for 36.3–64.1% of the total bacterial microbiota. The predominant species among Gram-positive cocci in all tested samples was *Staphylococcus xylosus*. The second most numerous bacterial group isolated from these kinds of samples was Gram-positive bacilli, constituting 24.7–28.2% of all identified strains. The most frequently isolated species of Gram-positive bacilli from all tested samples were *Bacillus licheniformis, Bacillus pumilus* and *Bacillus subtilis*. All these above-mentioned strains were frozen at −80 °C for analysis by RAPD-PCR in the next step of this study.

**Table 3:** Bacterial taxa isolated from the different types of samples collected at the power plant

Bacteria	Bioaerosol	Biomass	Respiratory masks	Swabs
**Gram-positive cocci**	**(20.6%)**	**(5%)**	**(64.1%)**	**(36.3%)**
*Aerococcus viridans*	×			
*Enterococcus faecalis*				×
*Kocuria kristinae*	×			
*Kocuria varians*				×
*Micrococcus* spp.	×		×	×
*Staphylococcus aureus*		×		
*Staphylococcus chromogenes*				×
*Staphylococcus epidermidis*			×	×
*Staphylococcus lentus*			×	×
*Staphylococcus sciuri*	×	×	×	×
*Staphylococcus simulans*			×	
*Staphylococcus* spp.	×	×		
*Staphylococcus xylosus*	×	×	×	×
*Streptococcus mutans*			×	
*Streptococcus pyogenes*			×	
*Streptococcus* spp.			×	
**Nonsporing Gram-positive rods**	**(12%)**	**(25.7%)**	**(2.8%)**	**(21.5%)**
*Arthrobacter* spp.		×	×	×
*Brevibacterium* spp.	×			
*Cellulomonas* spp.	×	×		×
*Corynebacterium auris*		×		
*Corynebacterium propinquum*			×	
*Corynebacterium* spp.	×	×		×
*Leifsonia aquatica*		×		
*Microbacterium* spp.	×	×		×
**Gram-positive bacilli**	**(39.8%)**	**(43.9%)**	**(28.2%)**	**(24.75)**
*Aneurinibacillus aneurinilyticus*			×	
*Bacillus circulans*	×	×		
*Bacillus firmus*	×	×		
*Bacillus lentus*		×		
*Bacillus licheniformis*	×	×	×	×
*Bacillus pumilus*	×	×	×	×
*Bacillus* spp.	×	×		×
*Bacillus subtilis*	×	×	×	×
*Brevibacillus laterosporus*	×			
**Gram-negative rods**	**(8.8%)**	**(13.2%)**	**(3.8%)**	**(11.7%)**
*Burkholderia cepacia*				×
*Chryseobacterium indologenes*				×
*Erwinia* spp.		×		
*Escherichia coli*			×	
*Pantoea* spp.	×	×		
*Proteus mirabilis*		×		
*Pseudomonas fluorescens*	×	×		
*Pseudomonas oryzihabitans*	×	×		×
*Pseudomonas* spp.				×
*Stenotrophomonas maltophilia*	×	×	×	
**Mesophilic actinomycetes**	**(18.8%)**	**(12.2%)**	**(1%)**	**(5.9%)**
*Actinomyces* spp.	×	×		
*Actinomyces radingae*		×		
*Nocardia* spp.				×
*Rhodococcus* spp.	×			×
*Streptomyces* spp.	×	×	×	

The percentage conttibutions to the total isolated bacterial microbiota of bacterial groups are given in brackets

### RAPD-PCR analysis

**Fig. 1:**
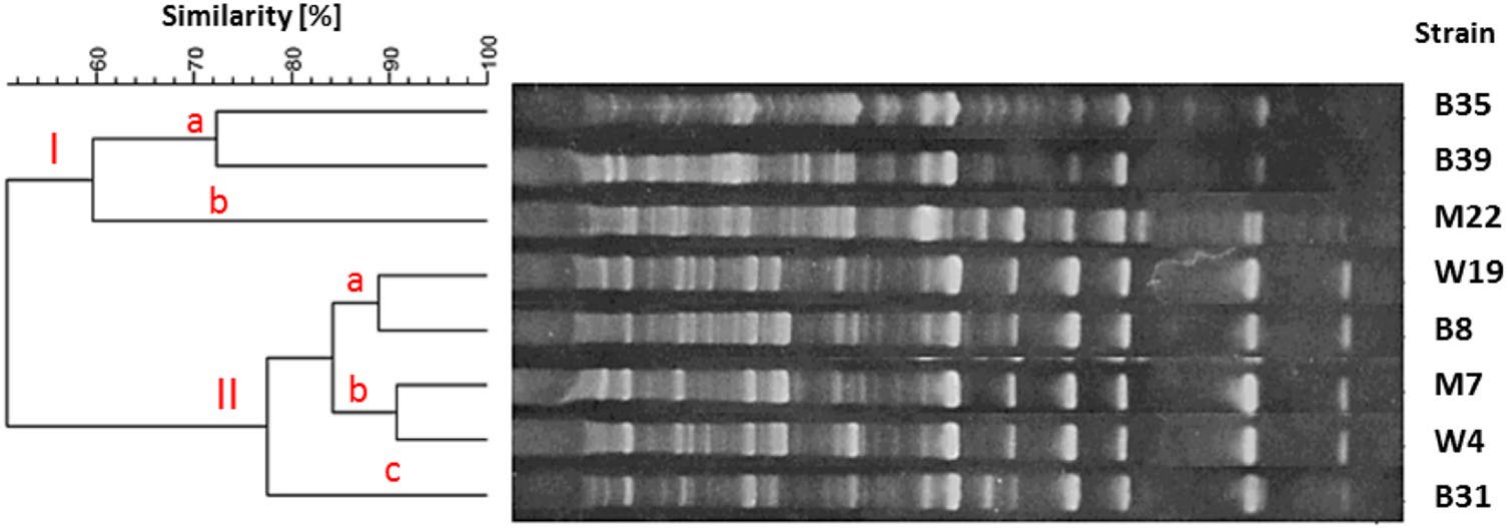
Dendrogram derived from RAPD-PCR profiles of *Bacillus licheniformis* generated with the Bac1 primer. *M* isolates from masks, *W* isolates from swabs, *B* isolates from biomass

**Fig. 2:**
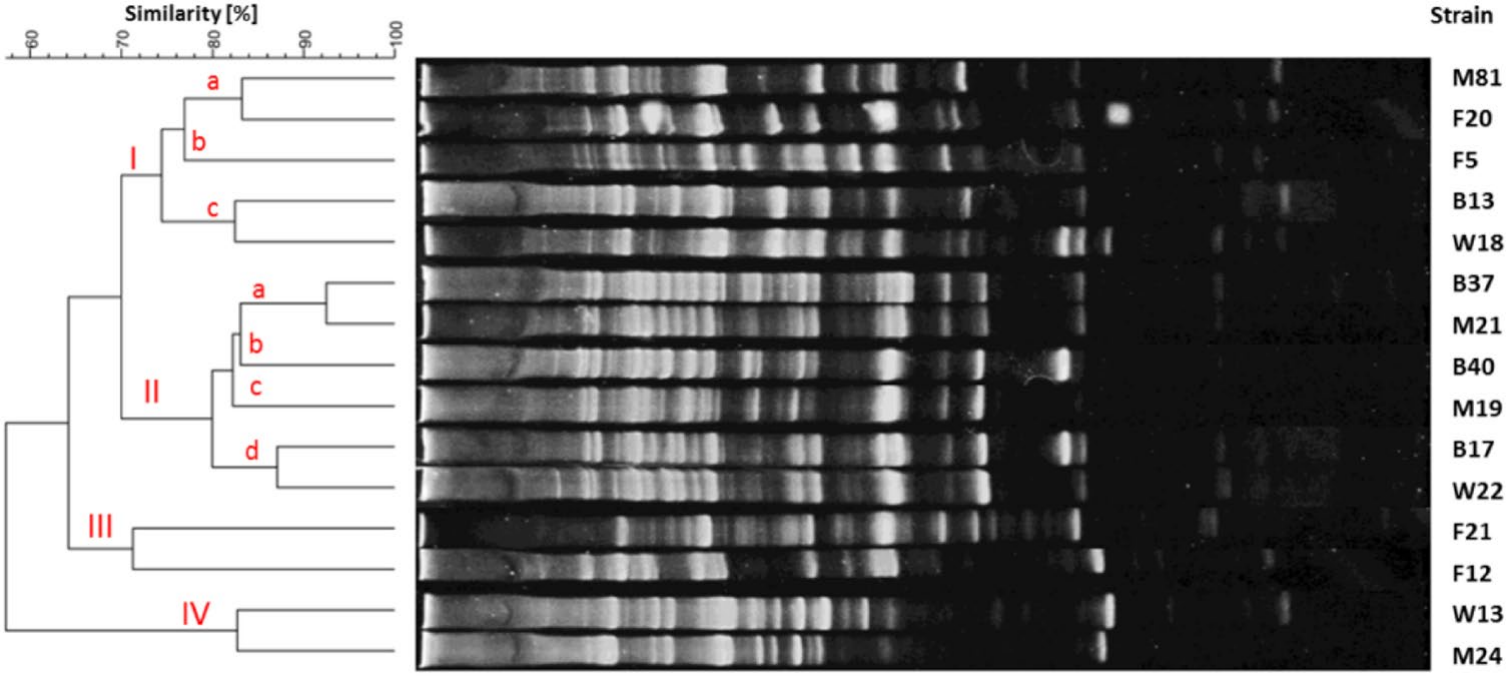
Dendrogram derived from RAPD-PCR profiles of *Bacillus pumilus* generated with the Bac1 primer. *F* isolates from bioaerosol, *M* isolates from masks, *W* isolates from swabs, *B* isolates from biomass

**Fig. 3:**
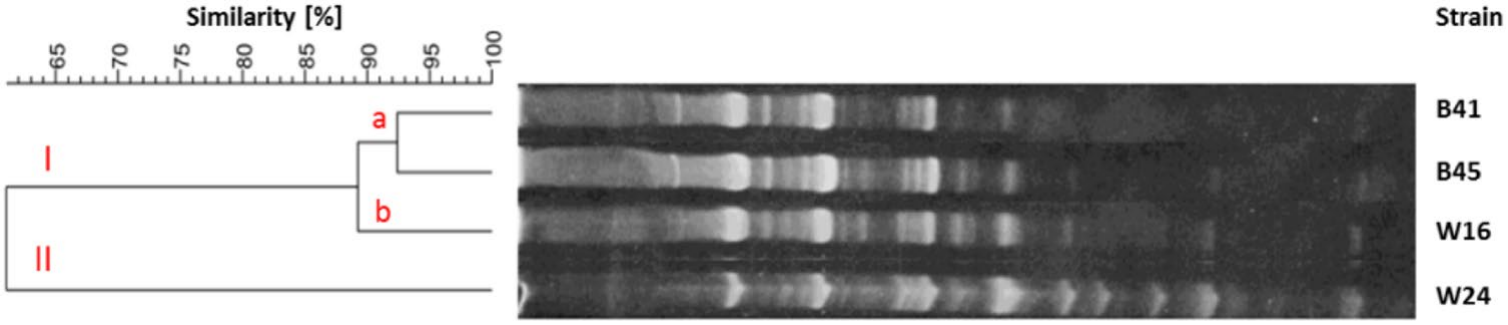
Dendrogram derived from RAPD-PCR profiles of *Bacillus subtilis* generated with the Bac1 primer. *W* isolates from swabs, *B* isolates from biomass

**Fig. 4:**
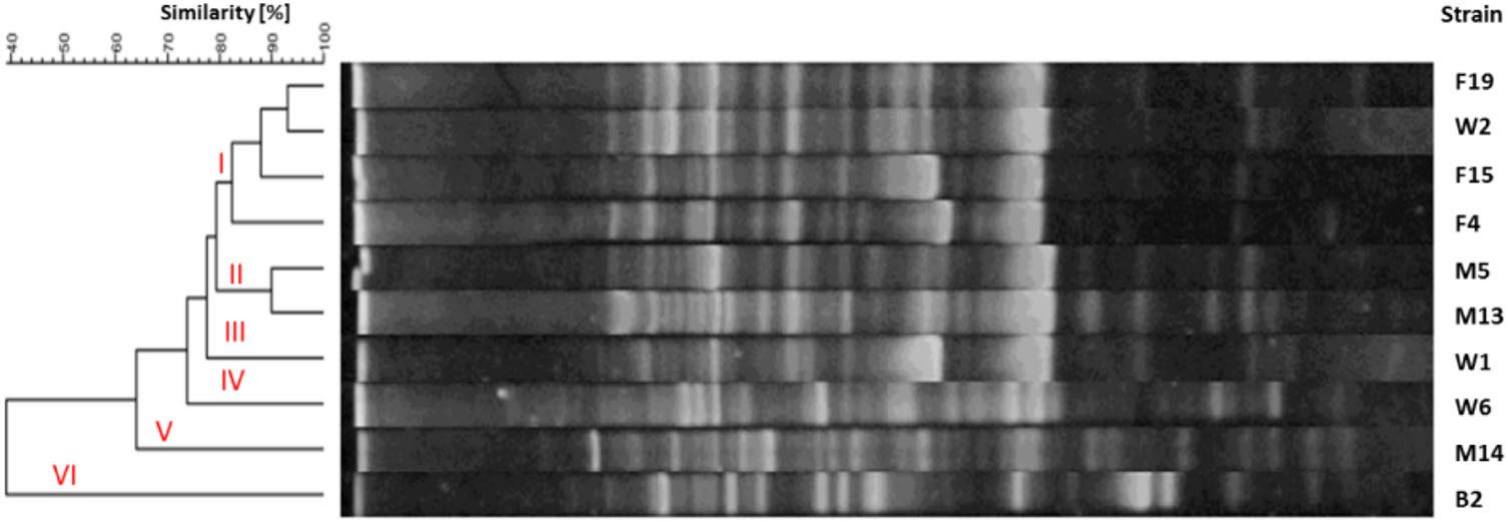
Dendrogram derived from RAPD-PCR profiles of *Staphylococcus xylosus* generated with the Xyl2 primer. *F* isolates from bioaerosol, *M* isolates from masks, *W* isolates from swab, *B* isolates from biomass

The products of RAPD-PCR reactions were electrophoretically separated and visualized according to an earlier described procedure. The length of the products ranged from 5,000 to <100 bp. The amount of PCR products allowed the intra-species differentiation of strains. The dendrograms for each species of tested bacteria are shown in Figs. 1, 2, 3 and 4. The genomic variability of *B. licheniformis* strains was reflected by RAPD-PCR analysis using “Bac1” primer (Fig. 1). Among isolates, two main clusters were identified at 60% similarity: I—grouped isolates from biomass and masks; II—grouped isolates from swabs, biomass and masks. Strains belonging to sub-clusters IIa (isolates from swabs and biomass) and IIb (isolates from masks and swabs from the hands) had similarity at the level of 88.9 and 90.7%, respectively. Such high similarity of these strains suggested their common source of origin (“close relationship”). Fifteen strains of *B. pumilus* were analyzed by RAPD-PCR using “Bac 1” primer (Fig. 2). Four major clusters were obtained at 65% similarity level: I—grouping strains isolated from all types of samples; II—grouping strains from hands, biomass and masks; III—grouping isolates from bioaerosol; IV—grouping strains from masks and swabs. Cluster I was divided into three sub-clusters: Ia (isolates from masks and bioaerosol), Ib (one isolate from bioaerosol) and Ic (isolates from biomass and swabs), which had a high genetic similarity level of 83.2, 79.4 and 82.5%, respectively. Cluster II included strains sharing the highest similarity level of 92.5% (IIa—samples of biomass and masks) and 87.1% (IId—biomass samples and swabs), which suggested their common origin. Fifteen strains of *B. subtilis* were analyzed by RAPD-PCR using “Bac 1” (Fig. 3). Two major clusters were obtained at the 60% similarity level. Cluster I grouped isolates from biomass and swab samples (88% similarity). The second cluster contained a single strain isolated from workers’ hands. Among the studied strains, two of them belonging to sub-cluster la (isolates from biomass) had a high genetic similarity level of 92.4%. Ten strains of *Staphylococcus xylosus* were analyzed by RAPD-PCR using the “Xyl2” primer (Fig. 4). The level of similarity between the isolates ranged from 24.9 to 92.5%. Six clusters were obtained above the 40% similarity level. Cluster I grouped isolates from bioaerosol and swab samples, and cluster II contained isolates from masks. Clusters III andVI grouped strains isolated from masks, swabs and biomass. The highest similarity level was observed in cluster I—between isolates from bioaerosol and swab samples (82.6–93.1%); and cluster II—between strains isolated from masks (90%).

## Discussion

The study showed that the work at a power plant utilizing biomass for energy production is associated with microbial exposure in a heavily contaminated environment. The mean concentration of bacterial aerosol at the workplaces reached the level of 1.2 × 10^6^cfu/m^3^ (standard deviation, SD, 1.4 × 10^6^cfu/m^3^). The concentrations noted were similar to those observed by other scientists studying this kind of occupational environment, e.g. Madsen (2006), Madsen et al. (2009), Rohr et al. (2015) and Laitinen et al. (2016). The mean concentration of bacteria in the biomass samples was 3.8 × 10^4^cfu/g (SD – 5 × 10^4^cfu/g). The qualitative analysis of biomass samples revealed that these raw materials were the primary source of bacteria at the examined workplaces. The observed levels and composition of bacterial microbiota were similar to those described by Ławniczek-Walczyk et al. (2012), Madsen et al. (2004), Sebastian et al. (2006) and Laitinen et al. (2016). The noted culturable microbial concentrations at workplaces exceeded the Polish OEL (occupational exposure limits) proposals (which are 1 × 10^5^ CFU/m^3^ and 2.0 × 10^4^ CFU/m^3^, for mesophilic bacteria and Gram-negative rods, respectively) in this type of working environment (Górny et al. 2011). Among all isolated culturable microorganisms, eight bacterial (*Actinomyces* spp., *B. subtilis, Corynebacterium* spp., *P. mirabilis, S. aureus, S. pyogenes, Streptococcus* spp. and *Streptomyces* spp.) species were classified as risk group 2, according to an Ordinance of the Polish Ministry of Health (2005), and as such may be responsible for adverse health outcomes in exposed individuals. The concentrations of mesophilic actinomycetes, *Corynebacterium* and Gram-negative rods were low. However, as a result of exposure to this type of microorganisms, among people susceptible to microbial allergens or those with weakened immune systems, adverse health effects such as allergic alveolitis, organic dust toxic syndrome and allergy may occur. Among the significant hazards to the health of exposed workers in the tested power plant are endotoxins from Gram-negative rods. Inhalation of endotoxins may result in acute inflammation in the respiratory tract and the development of diseases such as asthma, chronic bronchitis or *allergic alveolitis* (Dutkiewicz 1997). Bacteria from *Streptococcus* and *Staphylococcus* genera are members of the normal human microbiota. Some of them can also cause numerous skin and respiratory tract infections: e.g. pharyngitis and tonsillitis (*S. pyogenes*), boils, furuncles and impetigo (*S. aureus*) (Foster 1996; Patterson 1996). The observed prevalence of bacilli in all tested samples is directly related to their source of emission, which in this case is raw plant material. Similar domination of bacilli in the air samples was reported by Rohr et al. (2015) at biomass-fired power plants and by Dutkiewicz et al. (2001) at wood processing facilities. It has been documented that the bacteria of the genus *Bacillus* produce proteolytic enzymes that have allergenic properties (Dutkiewicz 1997). From the species listed in Table 3, *B. licheniformis, B. subtilis, B. circulans* and *B. pumilus* may be responsible for occasional respiratory, ear, eye and urinary tract infections in exposed individuals (Turnbull 1996).

To reduce the effects of exposure to biological agents at the examined workplaces, several technical and organizational measures were applied as well as personal protective equipment (including protective clothing, respirators and footwear) were used. Power plant workers were equipped with FFP2-type disposable respiratory masks. Masks were utilized in accordance with the manufacturer’s instructions and common occupational health and safety practices. If necessary, masks were replaced by workers with new ones—even two to three times a day, to provide the required safety and breathing comfort. In practice, it is not possible to determine the exact duration of the protective effect of the masks. It is understood that when the user feels an increase in breathing resistance (e.g. after clogging of the filter layer), the mask should be replaced with the new one (Majchrzycka and Brochocka 2008). The results of this project clearly indicate a very high level of microbial contamination of the filter surface of the masks. The analysis of masks samples revealed the presence of microorganisms, both typical physiological skin microbiota and from the human oral cavity, as well as strains that occurred in the biomass and in the air at the workplaces. Few studies conducted by Brosseau et al. (1997) have shown that microorganisms such as *Staphylococcus epidermis* and *Bacillus subtilis* caught on the filter surface of the masks can preserve their viability for a long time. It has been demonstrated that after 5 days of exposure to bioaerosols, *Staphylococcus* and *Bacillus* bacteria on masks showed culturability at the level of 61 and 98%, respectively. These results indicate that pathogenic microorganisms can survive and replicate on protective masks, and thus used masks may be an additional active source of infection at workplaces.

Another important element in reducing the spread of harmful microbiological agents in the workplace is hand hygiene of employees. The majority of the microorganisms isolated from human skin represent their physiological microbiota, such as Gram-positive cocci from *Staphylococcus* or *Micrococcus* genera and Gram-positive bacteria from *Corynebacterium* and *Brevibacterium*. The surface concentrations of these bacteria range from 4 × 10^4^ to 5 × 10^6^cfu/cm^2^ of the human skin. The dirty hands may be an excellent source of nutrition for many pathogenic microorganisms. Laboratory studies show that even a single contact with contaminated surfaces can transfer onto the skin 100–10,000 microbial cells (Kramer et al. 2006; Dzierżanowska-Fangrat et al. 2010; Scott 2013) In this study, the average concentration of bacteria in swab samples from workers’ hands was 1.4 × 10^4^cfu/ml (SD – 1.4 × 10^4^cfu/ml). As in the case of mask samples, the same strains as from biomass and bioaerosol were isolated from the swabs samples. It should be noted that many pathogens have an ability to survive on dry surfaces even for several months. Among the most easily transmissible bacteria to the skin are pathogens from the family *Enterobacteriaceae* (e.g. *Escherichia coli, Salmonella* spp.) and Gram-positive cocci such as *Staphylococcus aureus* (Kramer et al. 2006). Contaminated hands can also lead to re-contamination of other surfaces and materials with which the person had a direct contact. The results of this study suggest the urgent need for additional training among power plant workers on hand hygiene, as bad hygiene habits of employees can easily cause numerous adverse health outcomes including respiratory, skin and gastrointestinal tract infections. Moreover, education on the proper use of the personal protective equipment (and in particular how to properly put on and take off a disposable respirator) must be provided to all power plant workers.

The use in this study of a combination of traditional (macro-, microscopic and biochemical) and modern molecular methods allowed to obtain unique data on both the degree of workers’ exposure to biological agents at the workplace and the route of transmission of the most common bacteria associated with the processing of the biomass. In this study, 37 bacterial strains belonging to the genus *Bacillus* (27) and *Staphylococcus* (10) were isolated from the occupational environment and subsequently genotyped by the RAPD-PCR method. Analysis of the electrophoretic profiles and dendrograms obtained for each bacterial species showed significant genetic relationship between isolates derived from different sources. Among the analyzed *Bacillus* strains, 19 isolates showed a similarity level above 80%. In the case of *Staphylococcus xylosus* isolates, six exceeded 80% level of similarity. These results demonstrated that the biomass is the primary source of bacteria in the examined occupational environment. The molecular methods used confirmed the results obtained by traditional methods based on macro- and microscopic analysis and biochemical tests. The RAPD-PCR method showed close relationship between the isolates originating from individual workers and environmental samples. These results also illustrate that biomass-associated bacteria can be easily transferred to human hands and mask during working activities. To improve hygiene practices at the workplaces, adequate training courses for workers should be introduced as a key element of the prevention strategy. Analysis of the electrophoretic profiles of bacteria from *Bacillus* and *Staphylococcus* genera suggests that the major way by which these microorganisms spread is by direct contact with the biomass. The relationship between airborne bacterial strains was less pronounced. This may indirectly confirm the existence at the workplace of other permanent reservoirs of these bacteria, such as dirty work surfaces, machines, walls and floors of storage halls. It should be emphasized that the bacteria of the genus *Bacillus* can permanently colonize different surfaces due to of the ability to form spores (endospores). These bacteria are resistant to adverse conditions, such as a lack of nutrients in the environment, temperature changes, drying, some disinfectants and UV radiation (Silva et al. 2013). It was reported that *S. xylosus* (human opportunistic pathogen) may adapt to different environments by its capability to form a biofilm on biotic and abiotic surfaces such as glass, polystyrene and steel (Planchon et al. 2006). The observed similarity between RAPD profiles of the studied *Bacillus* and *Staphylococcus* strains isolated from biomass, masks and swabs may suggest a possibility of secondary contamination of hands and masks due to insufficient fulfilment of hygiene recommendations by the workers.

According to the best knowledge of the authors, the presented results of genetic diversity of bacterial strains isolated from occupational settings, related to processing of biomass, are the first of this kind in Poland and among few in the world (Adair et al. 2002). In this study, the method of RAPD-PCR belonging to the most universal genetic typing techniques was used. The RAPD-PCR method was successfully used for the identification and differentiation of many species of Gram-positive and Gram-negative bacteria and fungi (Alvarez-Perez et al. 2009; Bogiel and Gospodarek 2010; Chiang et al. 2014; Diab and Al-Turk 2011; Stefańska et al. 2008; Zeinali et al. 2015). However, in the available scientific literature, information on its use in the occupational environment is rather scarce. Perhaps, this is due to some limitations resulting from the lack of an adequate level of reproducibility of the results between different laboratories (Wolska and Szweda 2012). It should be noted, however, that using some guidance notes on the reaction conditions, it is possible to achieve a high repeatability of results. In this project, an optimization process of the RAPD-PCR reaction was carried out and successfully led to reliable results. Each step of the reaction was done under conditions compatible to the protocol (i.e. appropriate concentration of primer and template DNA, use of appropriate temperature profiles for PCR and stable conditions for electrophoresis) and with the use of the same reagents (in particular, the type of the thermostable polymerase used). In this type of PCR reactions, the primer length is usually from 10 to 25 pz and the G + C content is between 60 and 70% (Di Maria et al. 2002; Gajbhiye et al. 2007; Kumar and Gurusubramanian 2011; Vela et al. 2012; Wolska and Szweda 2012). In our study, the overall similarity to bacterial species ranged from 39 to 58%. The observed degrees of similarity are comparable or lower than those reported in other laboratory experiments (Cocconcelli et al. 1997; Gajbhiye et al. 2007; Morandi et al. 2010; Vela et al. 2012). The lower degree of similarity between the isolates could be because the analyzed strains were derived from different sources (i.e. from the environment and humans), and thus to a certain extent represented nominally greater genetic diversity. Another reason may also be the number of used primers. Perhaps, the use of several primers in the RAPD-PCR reaction could reveal more genetic links between the isolates. The obtained results clearly indicate the need to continue these studies, focusing e.g. on monitoring of bacterial pathogens in an occupational environment associated with biomass processing. They also pointed out the advisability of further use and evaluation of other genotyping methods to verify the above-described results and expand the current knowledge about the genetic diversity of microorganisms present at power plants and based also on the utilization of other energy biofuels.

## Conclusion

In summary, the use in this study of the RAPD-PCR typing method allowed the visualization of the relationship between the bacterial strains isolated from bioaerosol, biomass, protective masks and swabs from the hands of power plant workers. The presented results broaden our understanding of the biodiversity of microorganisms in the occupational environment associated with biomass processing. The PCR-based methods seems to be an good tool for a fast and precise typing of bacterial strains isolated from different sources in the occupational environment. Such methods may help to implement appropriate prophylactic procedures and minimize transmission of infectious agents at workplaces.
